# Fast, multicolour optical sectioning over extended fields of view with patterned illumination and machine learning

**DOI:** 10.1364/BOE.510912

**Published:** 2024-01-25

**Authors:** Edward N. Ward, Rebecca M. McClelland, Jacob R. Lamb, Roger Rubio-Sánchez, Charles N. Christensen, Bismoy Mazumder, Sofia Kapsiani, Luca Mascheroni, Lorenzo Di Michele, Gabriele S. Kaminski Schierle, Clemens F. Kaminski

**Affiliations:** 1 Department of Chemical Engineering and Biotechnology, University of Cambridge, Cambridge, CB3 0AS, UK; 2 fabriCELL, Molecular Sciences Research Hub, Imperial College London, London, W12 0BZ, UK

## Abstract

Structured illumination can reject out-of-focus signal from a sample, enabling high-speed and high-contrast imaging over large areas with widefield detection optics. However, this optical sectioning technique is currently limited by image reconstruction artefacts and poor performance at low signal-to-noise ratios. We combine multicolour interferometric pattern generation with machine learning to achieve high-contrast, real-time reconstruction of image data that is robust to background noise and sample motion. We validate the method *in silico* and demonstrate imaging of diverse specimens, from fixed and live biological samples to synthetic biosystems, reconstructing data live at 11 Hz across a 44 × 44*μm*^2^ field of view, and demonstrate image acquisition speeds exceeding 154 Hz.

## 1. Introduction

Widefield fluorescence microscopy permits the imaging of biological structures with a high specificity, however, out-of-focus light limits image contrast. Unless planar excitation profiles are used for illumination – such as in lightsheet [1] or HiLo [2] microscopy – fluorescent probes located above and below the focal plane contribute to the signal collected in the final image.

Scanning confocal microscopy circumvents this problem through the use of a pinhole to physically reject out-of-focus signal. While this is effective at increasing contrast, only a single point in the sample can be imaged at a time and images must be built up sequentially, pixel-by-pixel, greatly increasing acquisition time. Furthermore, high excitation powers are required to compensate for the signal lost through the pinhole.

A different approach to achieve optical sectioning (OS) is through structured illumination microscopy (SIM) [3]. Here, a fluorescent sample is illuminated by patterned excitation light and the emitted fluorescence is imaged with widefield detection. In super-resolution (SR) SIM, interference patterns are produced by the interaction of the patterned excitation with the structures of the sample and these interference patterns are used to extract high-resolution information about the sample [4,5]. OS-SIM makes use of the fact that the modulation depth of the excitation pattern is highest in the in-focus plane and decreases rapidly with distance from it. Hence, the in- and out-of-focus structures can be distinguished by differences in stripe contrast. To achieve this, the sample is typically illuminated with a sinusoidal stripe pattern, and three sequential images are acquired as the pattern is shifted in phase over the sample. Under these conditions, only the in-focus structures will show a change in intensity between the phase shifted images and therefore these can be extracted and the three raw images reconstructed into one optically sectioned image. This method was pioneered by Neil *et al.* [3] who used the squared difference (SD) between the three phases to reconstruct the image:

(1) $$I_{R}^{2}={\left(I_{1}-I_{2}\right)}^{2}+{\left(I_{2}-I_{3}\right)}^{2}+{\left(I_{1}-I_{3}\right)}^{2},$$

where $I_{R}$ is the reconstructed image and $I_{n}$ represents the $n^{th}$ phase image of the sample under striped illumination with the phase shifted by 2(n−1)π/3 . Phase stepping in this way ensures a uniform average illumination and prevents striping artefacts in the reconstructed image. While simple to implement, this method is highly sensitive to noise, which becomes amplified in the SD reconstruction. Additionally, even small movements of the sample or deviations from the ideal phase step size introduce reconstruction artefacts. Later efforts improved on this early SD method, by compensating for small errors in pattern phase [6] and reducing artefacts at low signal-to-noise ratios with HiLo filtering [7]. However, reconstructions remain sensitive to noise and prone to artefacts.

Since the early days of mechanically moved diffraction gratings, there have also been numerous developments in the techniques used to generate the sinusoidal patterned illumination. Currently, the most common techniques are based on digital micromirror devices (DMDs) [8] or liquid crystal spatial light modulators (SLMs) [9–11], with SLMs generally proving the more popular technology due to the improved light efficiency. When combined with suitable image denoising algorithms, the fast switching times of these devices have allowed for SR-SIM at speeds exceeding 180 Hz [12]. However, there are a number of limitations associated with these, including the complexity of the optical setup, the high cost of components with a sufficient optical flatness, and a field of view (FOV) that is limited by the physical size of the device.

Complications also arise when these methods are to be used for imaging in multiple colours. For optimal reconstructions of OS-SIM data, the spatial frequency of the striped illumination pattern must be half of the maximum spatial frequency observable by the microscope [3]. This resolution limit changes with wavelength, and since SLMs can only display a single pattern at a time, either colour channels must be imaged sequentially, or the user must accept a reduced performance. While the impact of using the same pattern for different wavelengths is less severe for OS-SIM than for SR-SIM, problems arise when a large range of wavelengths is required. In particular, the desired diffraction orders for the different colours become spatially separated to such an extent that using a mask to filter out spurious higher diffraction orders becomes challenging. When using liquid crystal SLMs, there are further limitations in the range of illumination wavelengths that can be used, as the diffraction efficiency drops rapidly outside of the designed centre wavelength and the devices themselves become damaged by high-power illumination with shorter wavelengths.

We address these issues by combining machine learning (ML) with interferometric pattern generation for OS. Our interferometric SIM method is low cost, simple to implement, and is ideally suited to high speed imaging of samples with a wide range of excitation wavelengths. We use two complementary neural networks to reconstruct OS-SIM data: a fast network for real-time reconstructions and a heavier network for post-acquisition reconstructions. Using the fast-reconstruction network, we demonstrate on-the-fly processing of OS-SIM data, allowing users to immediately and intuitively visualise reconstructions as they navigate the sample. We also demonstrate that by building sample motion into the image formation model, we remove the striping artefacts seen when imaging highly dynamic samples. The software to achieve these reconstructions is packaged in a user-friendly graphical user interface (GUI) which makes the method immediately applicable to both new and existing OS-SIM setups.

## 2. Methods

### 2.1 Hardware implementation

**Fig. 1.**
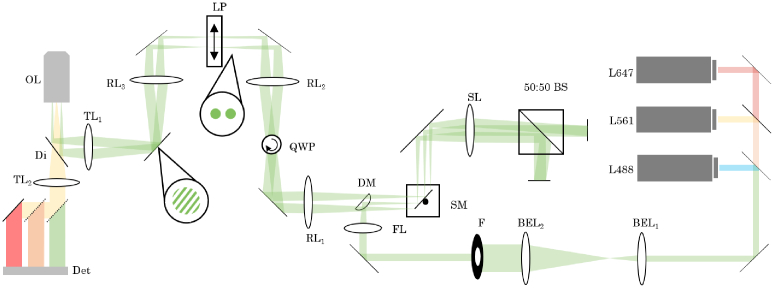
Optical layout for interferometric SIM pattern generation [13]. Three laser lines (**L**) with wavelengths 488 nm (Cobolt Calypso, 200 mW), 561 nm (Oxxius SLIM-561-150, 500 mW) and 647 nm (Toptica iBeam SMART, 100 mW) are combined coaxially and the beam is then expanded and collimated by two lenses (**BEL_1,2_**) to flatten the intensity across the field of view. A field stop (**F**) restricts the excited area of the sample to minimise unwanted photodamage. The excitation beam is focused by a focusing lens (**FL**, Thorlabs, AC254-150-A) and reflected by a D-shaped mirror (**DM**, Thorlabs, PFD10-03-F01) onto a galvanometric scan mirror (**SM**, Scanlab, dynAXIS-M) which directs the beam through a scan lens (**SL**, Thorlabs, AC508-150-A) and into the Michelson interferometer. The interferometer comprises a 2-inch 50:50 beam splitter cube (**BS**, Thorlabs, BS031) and a pair of ^1^/_2_-inch mirrors (Thorlabs, BB05-E01) mounted on micrometer translation stages (Thorlabs, XRN25C/M). The beamlets returning from the interferometer are descanned by the scan mirror and relayed to the microscope by a series of 150 mm focal length relay lenses (**RL_1−3_**, Thorlabs, AC508-150-A). Polarisation is controlled by a quarter wave plate (**QWP**, Thorlabs, AQWP05M-600) and linear polariser (**LP**). The two beamlets are then directed into the inverted microscope frame (Olympus, IX73) and onto the back focal plane of the objective lens (**OL**, Olympus, UPLSAPO60XW) through a tube lens (**TL_1_**, Thorlabs, TTL200A). The beamlets are focused by the objective so that they interfere in the sample plane to form a sinusoidal illumination pattern. Fluorescence signal is isolated with a quadband dichroic mirror (**Di**, Chroma, 405/488/561/647) and focused onto the detector (**Det**, PCO, edge 4.2bi). The fluorescence signal is separated into emission bands by two long-pass dichroic mirrors (Chroma, T635LPXR-UF2 and T560LPXR) enabling multiple colour channels to be imaged side-by-side on the detector.

To overcome the issues associated with SLMs and DMDs, we use an interferometric method for pattern generation (Fig. 1) [13]. The setup makes use of a Michelson interferometer to produce the sinusoidal illumination, where phase stepping is achieved by laterally sweeping the pattern across the sample with a single galvanometric mirror element. In this way, the periodicity of the sinusoidal interference fringes depends on the wavelength of the excitation light, meaning the fringe spacing is optimised for all wavelengths simultaneously. Additionally, interference fringes can be generated for any wavelength supported by the beamsplitting element. In our setup, fluorescence signal from the sample is collected and split into three colours using an image splitting device, permitting the simultaneous imaging of multiple colour channels. A *Python* GUI enables hardware control and display of reconstructed images in real time (Fig. S3). All source code is available in a GitHub repository [14].

### 2.2 Subtractive reconstructions

In addition to the classical SD approach (Eq. (1)), we tested a corrected SD method (Eq. (2)) [6] and a filtered SD reconstruction algorithm by Li *et al.* (Eq. (3)) [7]. The corrected SD method compensates for uneven phase stepping of the pattern by weighting the components of the reconstruction according to the phase of the excitation pattern:

(2) $$I_{corr.}^{2}=(I_{1}-I_{2})+{\left[\frac{\left(I_{2}-I_{3}\right)}{\tan\left(\frac{\phi_{2}-\phi_{3}}{2}\right)}+\frac{\left(I_{3}-I_{1}\right)}{\tan\left(\frac{\phi_{3}-\phi_{1}}{2}\right)}\right]}^{2}$$

where $I_{n}$ are the raw images and $\phi_{n}$ are the corresponding relative phases of the excitation pattern, calculated using the inverse matrix approach from Cao *et al.* [15].

Based on the HiLo concept introduced by Lim *et al.* [16,17], the mixed filtering method further refines this corrected OS-SIM image by combining the high spatial frequency information from the predicted widefield image, with the low spatial frequency information from the OS-SIM image. The widefield image is high-pass (HP) filtered and mixed with the low-pass (LP) filtered OS-SIM image according to a weighting parameter *α*:

(3) $$I_{filtered}=\alpha \cdot \text{HP}\left(I_{widefield}\right)+(1-\alpha )\cdot \text{LP}\left(I_{corr.}\right)$$

This has the effect of simultaneously removing the out-of-focus light while making use of the widefield image to reduce the noise introduced by the SD-SIM reconstruction. The filter width is determined by the frequency of the fringe pattern and the mixing parameter, *α*, is chosen based on the modulation depth of the pattern. Finally, the mixed filter method applies a stripe suppression filter to the reconstruction by attenuating the spatial frequencies in the image near to the spatial frequency of the excitation stripe pattern.

The basic, corrected and filtered SD reconstructions were calculated in *MATLAB*.

### 2.3 Machine learning reconstruction

Machine learning (ML) has become a powerful tool for the pre-processing, post-processing and reconstruction of SR-SIM data [20]. Compared to classical methods, ML can increase reconstruction quality and does not require the estimation of system parameters. For OS, ML has been used to generate sectioned images directly from widefield images [21]. By training a network on a sample-by-sample basis, learned *a priori* knowledge of the sample can be used to improve contrast, although this method is susceptible to sample variations and requires an existing optical sectioning system to provide the training data. ML has additionally been applied to the reconstruction of OS-SIM data [22], although the impacts of pattern phase error and sample movement have not been fully addressed. Here, we leverage recent advances in ML and optimise two distinct neural networks specifically for the reconstruction of interferometrically generated OS-SIM data. By using two networks, we are able to achieve real-time reconstructions at the point of acquisition, and high-fidelity reconstructions post-acquisition, yielding optimised results even for moving samples.

#### 2.3.1 Residual channel attention network

**Fig. 2.**
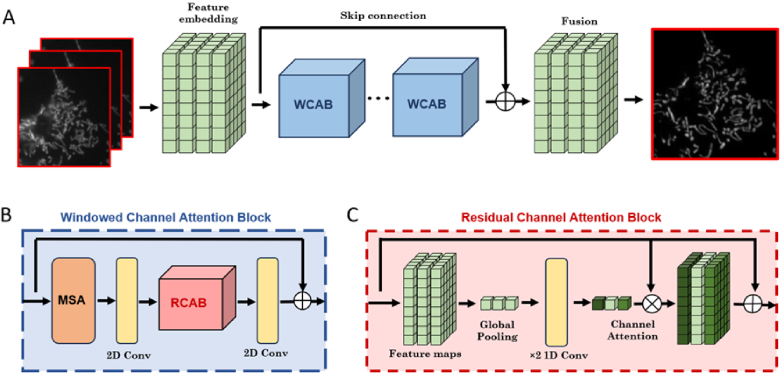
Network architectures for machine learning reconstructions. A: Architecture for the video super-resolution (VSR) based network. The initial data is embedded into a multi-dimensional feature map which is then passed through a sequence of five windowed channel attention blocks (WCABs) and fused into a single reconstructed frame. B: Each WCAB consists of a sequence of Swin transformer layers with multi-head self attention (MSA) [18] followed by a residual channel attention block (RCAB) [19]. C: Each RCAB comprises a channel attention mechanism using global pooling and two convolution layers to calculate weights for the input channels. A residual connection is then added to ensure the continuity of low-frequency information through the network.

In the first instance, ML-OS-SIM reconstructions were performed using a lightweight convolutional neural network (CNN) based on the residual channel attention network (RCAN) architecture [19,23]. This RCAN model has a low memory footprint and can perform reconstructions on low-end consumer graphics cards at rates compatible with live reconstruction during imaging. The network consisted of three groups of ten residual channel attention blocks (RCABs) each with 96 filters based on a 3 × 3 kernel (Fig. 2(C)). Unlike in previously reported implementations of the RCAN architecture for SIM, the initial "head" layer was adjusted to use learnable filters with a size of 7 × 7 .

#### 2.3.2 Video super-resolution network

For high-fidelity image reconstruction post-acquisition, we use a video super-resolution (VSR) transformer network based on the shifted window architecture (Fig. 2) [18,24–27]. By using 3-dimensional patches, this VSR network is optimised to combine information from adjacent frames in a sequence acquisition, offering improved performance on moving structures. The multi-head self-attention mechanism of the Swin transformer is supplemented with a channel attention mechanism based on a single RCAB (Fig. 2(C)).

#### 2.3.3 Data generation

To mitigate the challenges posed by training on experimentally acquired OS-SIM data, we simulated large datasets and employed transfer learning to train the network. This approach offers several advantages: firstly, for supervised learning, the ground truth is known, ensuring that the networks do not learn to generate the same artefacts produced by classical reconstruction algorithms. Secondly, large and diverse training datasets can be generated, enabling the models to learn the reconstruction process without overfitting to smaller experimental datasets. Thirdly, it is possible to train the network to be robust to the specific challenges associated with reconstructing interferometric OS-SIM data, particularly noise and sample motion.

Two separate datasets were generated for the network training with the datasets tailored for each network. For the RCAN model, where reconstruction speed requires a smaller network, the in-focus plane was simulated from a library of high-resolution static images. This aligns with the primary goal of the RCAN model: to provide the user with real-time reconstructions at the expense of robustness to the artefacts associated with moving samples. For the VSR network, these static OS-SIM data were supplemented with data simulating samples with moving structures. Here, the in-focus plane was generated from three sequential frames in video sequences from BBC nature documentaries, mimicking samples that move during acquisition. For both the VSR and RCAN training datasets, the structures of the out-of-focus planes of the sample were simulated by taking static images from the DIV2K dataset [28]. From these ground-truth images, model OS-SIM data were generated by multiplying both the in-focus and out-of-focus planes with a sinusoidal excitation pattern, and subsequently blurring them by convolution with either the in-focus or out-of-focus point spread functions (PSFs). The planes were then merged using a weighted addition. Varying levels of Gaussian and Poissonian noise were added to the raw frames after combination. The parameters for generating the PSFs and the excitation pattern were randomised, allowing the models to generalise to data collected on a range of microscopes and in varying imaging conditions.

#### 2.3.4 Network training

Both networks were trained in *Python* using the *Pytorch* framework. The networks were trained on 5000 simulated images for 200 epochs on an Nvidia RTX3070 graphics card. For the VSR network, these 5000 simulated images were composed of 2000 static targets and 3000 moving targets. The networks were trained using the Adam optimiser. For the initial 100 epochs, the mean square error (MSE) loss function was used, which was changed to the L1 absolute difference loss function for the final 100 epochs. These loss functions were calculated relative to the high-resolution ground-truth in-focus image. For the VSR network, the second frame in the sequence was used to provide this ground truth. Under these conditions, network training took approximately 20 h for the RCAN model and 38 h for the VSR network.

## 3. Results and discussion

### 3.1 Machine learning improves contrast and minimises noise in optical sectioning

**Fig. 3.**
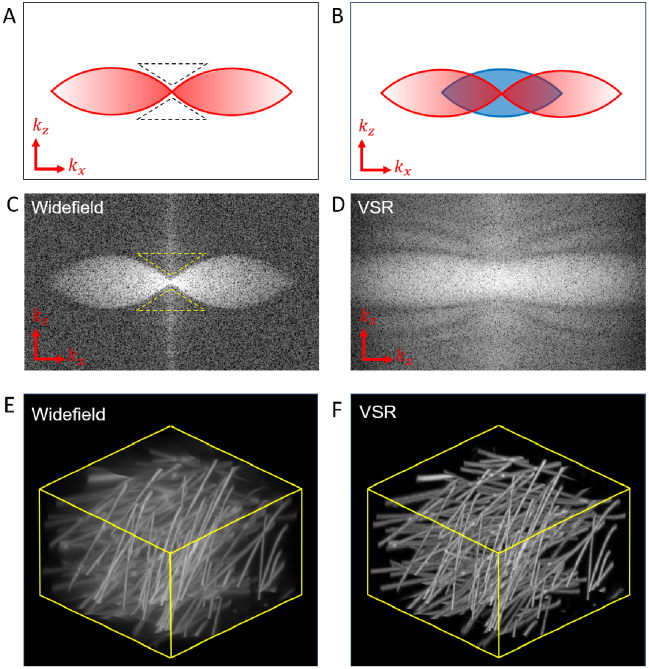
ML reconstruction of OS-SIM data recovers axial resolution and fills the missing cone. A: Schematic indicating the shape of the optical transfer function (OTF) of a widefield microscope in the $k_{x},k_{z}$ plane. B: Ideal OTF after OS-SIM reconstruction. Upon illumination with a stripe pattern, the low spatial frequency components (blue area) can be extracted and allocated to the correct location in frequency space. C: $k_{x},k_{z}$ projection of the widefield OTF from simulated OS-SIM imaging of point sources. The bright area indicates the supported spatial frequencies. In the $k_{z}$ direction, a cone of frequencies (yellow dashed line) is missing. These missing frequencies contain the axial information of the sample and this loss of information results in the poor background rejection seen in widefield imaging. D: Calculated OTF of VSR reconstruction of simulated OS-SIM images. Compared to C, the missing cone has now been filled, indicating that axial information has been recovered in the reconstruction. E: 3D perspective of simulated widefield imaging of a filament mesh. F: 3D perspective of VSR reconstruction of a simulated filament mesh.

**Fig. 4.**
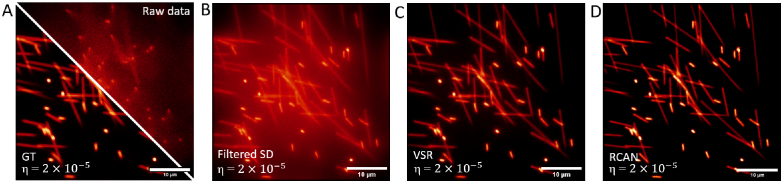
Machine learning reconstructions, VSR and RCAN, outperform square difference (SD) reconstructions at low signal-to-noise ratios. A: Comparison of the simulated ground truth (GT) confocal image, a maximum intensity projection of 50 slices from the simulated 3D volume with no Poissonian noise added, and the raw data: the expected widefield image from a single frame, calculated as the mean of the 3 OS-SIM frames. B-D: Maximum intensity projections of the filtered SD, VSR and RCAN reconstructions, taken from 50 slices with a noise level $\eta =2\times 10^{-5}$ . Scale bars = 10 *μm*.

**Fig. 5.**
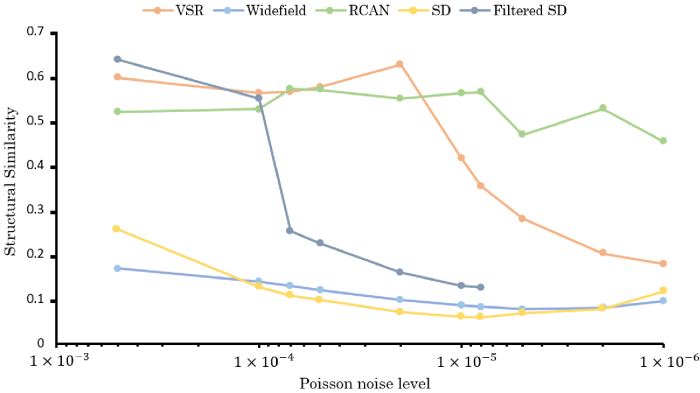
Error analysis of OS-SIM reconstruction methods. All methods decrease in performance as the signal level (Poisson factor, η ) decreases. At the noise levels tested, filtered SD outperforms basic SD at all levels and marginally outperforms VSR and RCAN reconstructions at the lowest noise levels. As noise increases, filtered SD shows a sharp drop in performance at $\eta =1\times 10^{-4}$ , vs $\eta =2\times 10^{-5}$ for the VSR approach. On these static samples, RCAN continues to perform reconstructions of comparable quality at levels down to $\eta =5\times 10^{-6}$ .

The models were initially validated by demonstrating their performance on simulated 3D samples comprising a mesh of filaments (Fig. 3). Simulated data were generated in *MATLAB* from an artificial ground truth by multiplying the mesh with a striped excitation pattern and convolving the fluorescent response with an ideal widefield PSF. The PSF was calculated using the Born and Wolf model for an emission wavelength of 600 nm, a water immersion objective lens with NA=1.2 , and voxels measuring $86\;\times \;86\;\times \;86\;{\text{nm}}^{3}$ . The recovery of lost axial information can be visualised in the Fourier transforms of the simulated and reconstructed data (Fig. 3). To measure the performance of the ML reconstructions in the presence of noise, a fixed level of Gaussian noise and varying levels of Poissonian noise were added to mimic imaging with low photon counts. Poissonian noise was modelled by sampling pixel values from a Poisson distribution scaled by $\eta \;\times \;10^{12}$ , where lower values of η correspond to fewer photons collected from the sample. Figure 4 shows a comparison of the ML and filtered SD reconstruction methods. The performance was further quantified by measuring the structural similarity between the ideal, noise-free, optically sectioned image and the reconstruction (Fig. 5). As expected, the fidelity of all reconstruction methods is reduced as noise is increased. However, ML reconstruction showed a lower sensitivity to noise, and VSR successfully reconstructed OS-SIM data with a fifth of the signal required for SD reconstruction.

### 3.2 Video transformer reconstruction removes motion artefacts from optical sectioning reconstructions

**Fig. 6.**
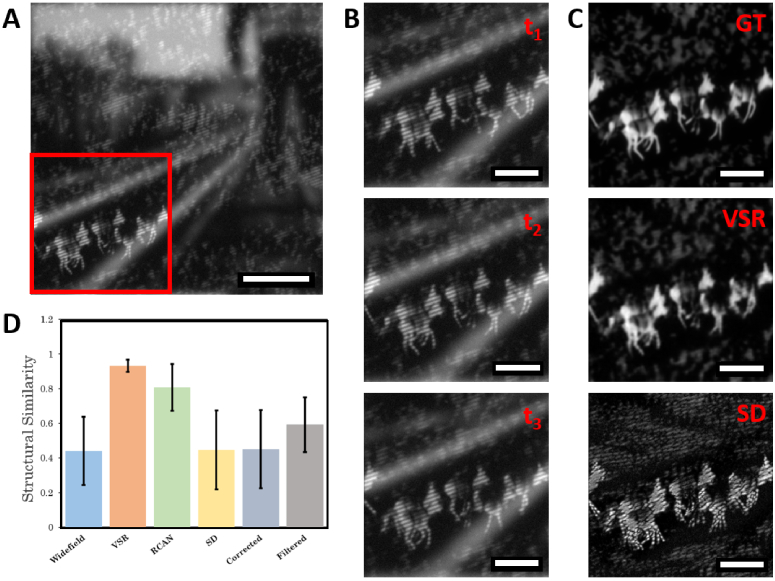
Comparison of reconstruction methods on dynamic OS-SIM data. A: Single frame from simulated imaging of a dynamic sample. Scale bar = 10 *μm*. B: Magnified view of the area indicated in A. The three input frames mimic a moving structure under shifting patterned illumination. C: (Top to bottom) ground truth (GT) ideal sectioning image; video super-resolution (VSR) reconstruction; squared difference (SD) reconstruction. Scale bar = 5 *μm*. D: Structural similarity (SSIM) scores for reconstruction methods averaged across eight reconstructions. Scores were calculated as the similarity between the reconstructed image and a model optically sectioned reconstruction GT. Error bars indicate the standard deviation in SSIM across all reconstructed images.

When using subtractive methods to determine variations in pattern contrast, it is vital that the sample does not move by more than the diffraction limit between pattern shifts. If this assumption is not satisfied, moving objects appear replicated and stripe artefacts are introduced into the final image. To measure the impact of sample movement on reconstruction quality, reconstruction methods were tested on data simulating OS-SIM imaging of structures that move between frames (Fig. 6(A),B). In the subtractive SD reconstruction, the moving structures produce the typical shadowing artefacts seen in OS-SIM imaging of highly dynamic samples (Fig. 6(C)). These artefacts, however, are effectively suppressed in the VSR reconstruction, which shows no shadowing or striping whilst still removing out-of-focus light from the images. This improved performance can be quantified by measuring the structural similarity between the reconstructions and an ideal image of the sample at a single timepoint during acquisition (Fig. 6(D)). This ideal ground truth was calculated from the second frame of the video data that was used to simulate the moving object (Fig. 6(B)). Averaged over 8 simulated images, the VSR method provided the most accurate and consistent reconstructions. It was observed that the increased variation in reconstruction quality seen with the other methods resulted from differences in the size and speed of the moving objects in the sequence, with reconstruction quality decreasing as movement in the sample increased. It should also be noted that, as with noisy data, the artefacts introduced by the subtractive methods can lead to reconstructions that show no quantitative improvement over widefield images, even if there is a qualitative improvement in image contrast.

### 3.3 Machine learning outperforms subtractive reconstruction on experimental optical sectioning data

**Fig. 7.**
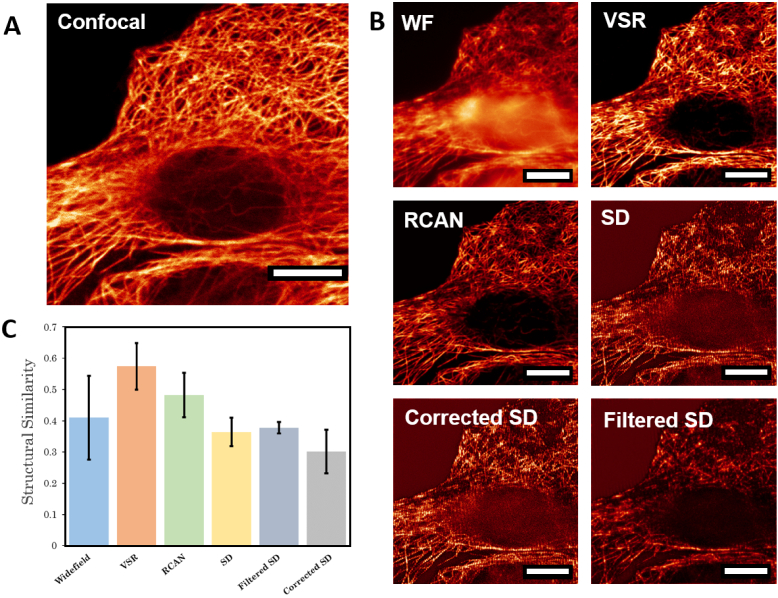
Comparison of optical sectioning structured illumination microscopy (OS-SIM) reconstruction methods to scanning confocal imaging. Machine learning (ML) outperforms classical methods and has comparable performance to confocal. A: Scanning confocal image of immunostained β -tubulin in fixed Vero cells. B: Comparison of widefield (WF) imaging to ML-OS-SIM reconstruction methods - video super-resolution (VSR) and residual channel attention network (RCAN) - and traditional reconstruction techniques: square difference (SD) [3], filtered SD [7] and corrected SD [6]. All techniques show an improvement in contrast and background rejection, however, classical methods are heavily impacted by noise, as seen in the top left corner of the images. In the SD reconstruction, the noise is amplified and the details of the sample are obscured. The filtered SD reconstruction removes this noise but at the expense of artefacts being introduced into the reconstruction. In comparison, the two ML-OS-SIM reconstructions successfully remove both the background and noise. C: Structural similarity index of the reconstructions to the corresponding confocal image averaged over six regions of the sample. Error bars show standard deviation in the similarity scores. All scale bars = 10 *μm*.

Finally, the reconstruction methods were validated on experimental data by imaging the same sample with confocal and OS-SIM microscopy. The confocal image provides an ideal ground truth reference to determine the reliability of the reconstructed images (Fig. 7). As expected, all OS-SIM methods qualitatively showed a reduction in the out-of-focus signal. However, quantitative analysis of the reconstructions reveals that for the subtractive methods, the amplified noise significantly reduces the reliability of the reconstruction, indicating that at even modest noise levels, reconstructions are a worse representation of the underlying structure than the widefield image. In contrast, both ML methods provided improved contrast and a more accurate representation of the sample.

### 3.4 ML-OS-SIM can image faster than point scanning confocal microscopy with similar imaging performance

**Fig. 8.**
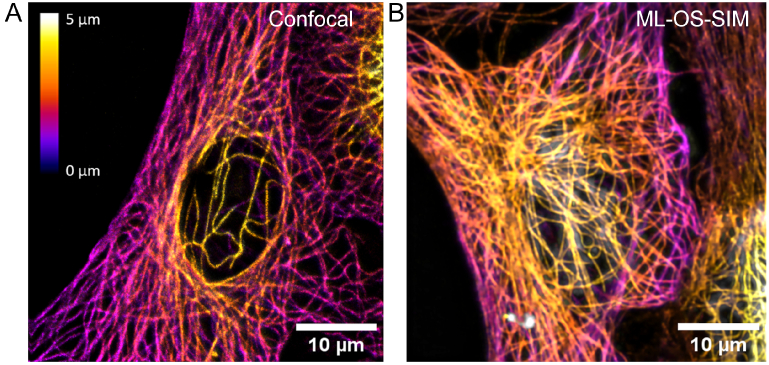
Machine learning optical sectioning structured illumination microscopy (ML-OS-SIM) produces optically sectioned volumes equivalent to scanning confocal microscopy at faster imaging speeds. Images show immunostained β -tubulin in fixed Vero cells imaged with A: deconvolved scanning confocal microscopy and B: ML-OS-SIM. Both techniques enable the 3D structure of the microtubule cytoskeleton to be resolved. Using ML-OS-SIM, the complete equivalent volume could be imaged and the reconstruction displayed to the user in < 2 s with a 50 ms exposure per frame compared to 2 min 36 s for scanning confocal microscopy. Timings were chosen to achieve similar sectioning performance. Colourmap indicates the z position. Scale bars = 10 *μm*.

To demonstrate the speed improvement possible with interferometric ML-OS-SIM, the system was compared to point scanning confocal microscopy (Fig. 8). The camera exposure time was adjusted to produce reconstructions of a similar quality to confocal images of the same sample. In both images, background light is effectively removed and the 3D microtubule network of the cell can be resolved free from artefacts. However, ML-OS-SIM imaging was considerably faster: a 44 × 44 × 5 *μm*^3^ volume could be imaged and the OS reconstruction presented to the user in < 2 s, whereas scanning confocal imaging took 2 min 36 s. That is, ML-OS-SIM was approximately 78 times faster. This speed advantage, in combination with the lower illumination laser power required, results in less photobleaching and photodamage to delicate samples (Supplement 1). An advantage of ML-OS-SIM is that a single detector can be used in conjunction with detection-splitting optics, whereas a confocal system would require an additional detector for each channel.

For single-colour imaging, live reconstruction data can be presented to the user at up to 11 Hz over the full FOV. Imaging can also be performed over smaller FOVs without live reconstruction when speed is important, enabling imaging speeds of 463 Hz over an 11 × 11 *μm*^2^ FOV, equivalent to 154 reconstructed frames per second. We note that at smaller FOVs and with a sufficiently bright sample, imaging speeds exceeding 4.8 kHz would be possible with the current interferometric setup (Supplement 1).

### 3.5 ML-OS-SIM enables the volumetric imaging of structures in multiple colours

**Fig. 9.**
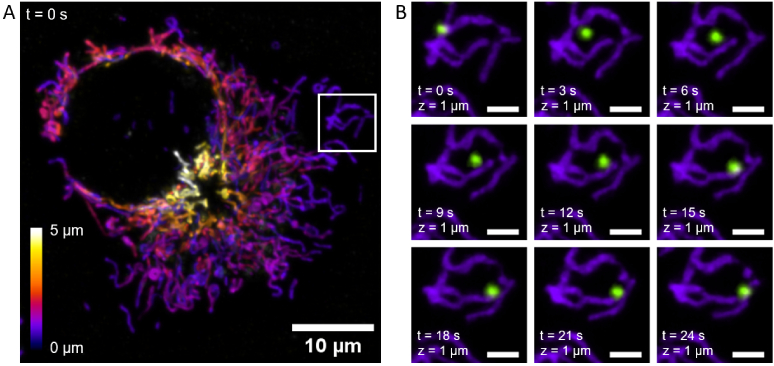
Machine learning optical sectioning structured illumination microscopy (ML-OS-SIM) enables video-rate mapping of live COS-7 cells, labelled with MitoTracker Orange and SiR Lysosome and excited simultaneously with the laser lines λ=561 and 647nm . A: Single-channel volume projection of the mitochondrial network where the colourmap indicates the z position. Scale bar = 10 *μm*. B: Dynamic interaction of lysosomes (green) with mitochondria (purple). Image sequence shows a projection of 3 adjacent slices from the data. Scale bars = 2 *μm*.

To demonstrate the capabilities of the technique for biological imaging, live cells were imaged in multiple colours across a 5 *μm* axial range (Fig. 9). Figure 9(A) depicts a maximum intensity projection of the mitochondrial network of a live cell imaged at a snapshot in time where the colourmap indicates the depth within the cell. No movement or striping artefacts are discernible in the image. Furthermore, the multicolour capability of the system allows several structures to be visualised without a temporal delay between colour channels. Figure 9(B) shows a two-channel time series of projections from the volumetric data. Here, a lysosome (green) can be seen to push a mitochondrial structure (purple) out of the way as it is transported through the cell.

### 3.6 Multicolour 3D imaging of DNA-nanotechnology biomimetic behaviours with ML-OS-SIM

**Fig. 10.**
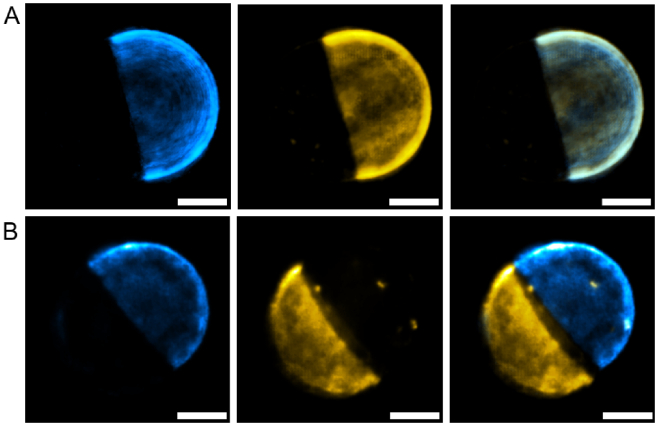
Optical sectioning and multicolour interferometric structured illumination enable fast, high-contrast 3D imaging of the lateral re-organisation of membrane-bound DNA nanostructures in Giant Unilamellar Vesicles (Fig. S5) [29]. Images are 3D projections of volumetric data. A,B: Representative vesicles before (A) and 12 h after (B) fueling a biomimetic cargo transport pathway with nucleic-acid signals. A: In the initial configuration, the DNA (blue) and liquid disordered phase (yellow) overlap, indicating the preferential affinity of DNA nanostructures for liquid-disordered phases. B: Cargo transport relocates the fluorescent nanostructures to the liquid-ordered phase. ML-OS-SIM enables samples to be imaged in multiple colours simultaneously, preventing motion artefacts and temporal offsets in the reconstructions. Scale bars = 5 *μm*.

In addition to biological samples, the contrast enhancement afforded by our ML-OS-SIM strategy enables the visualisation of processes in synthetic cell science. Recently, the synergy between lipid-membrane phase behaviour and the tools of DNA nanotechnology has gained traction to engineer bio-inspired responses in cell-like objects [30,31]. Figure 10 shows ML-OS-SIM images of DNA nanostructures tethered to phase-separated lipid membranes. The fast ML-OS-SIM technique allows for the reconstruction of multicolour 3D views of DNA-functionalised giant vesicles that can sustain biomimetic cargo transport pathways across their membrane surface (Fig. 10) [29]. Chemical stimulus, in the form of DNA oligonucleotides, triggers the lateral re-organisation of DNA nanostructures, leading to the transport of fluorescently labelled DNA cargoes (blue), away from the liquid-disordered phase (yellow) to liquid-ordered lipid domains (Fig. S5). The responsive DNA nanostructures harness established strand displacement mechanisms [32,33] and the preferential tendency of different hydrophobic anchors in membrane-bound DNA to enrich distinct lipid domains [29]. The speed of ML-OS-SIM enables 3D imaging of the structures, allowing for better visualisation and understanding of the re-organisation process [29].

## 4. Conclusion

To date, OS-SIM has been simultaneously limited by a lack of robust reconstruction algorithms and limitations in the instrumentation used for pattern generation. Here, we demonstrate that ML reconstruction methods enable multicolour OS-SIM across a 44 × 44 *μm*^2^ FOV, with the option to view real-time reconstructions at up to 11 Hz. The use of a shifted window transformer architecture makes our method immune to the reconstruction artefacts common in traditional, subtractive techniques.

Additionally, we demonstrate that the improved reconstruction methods are ideally combined with interferometric pattern generation. This method provides a simple and lower cost alternative to existing technologies based on SLMs. We achieve multi-label volumetric imaging, allowing for the interactions between structures to be visualised with no temporal lag. The interferometric pattern generation technique opens up the possibility of performing optical sectioning simultaneously over wavelength ranges spanning ultraviolet to far red with no changes to the optical setup. The widefield detection method employed by ML-OS-SIM means that optically sectioned images comparable to confocal microscopy can be achieved at speeds up to 154 Hz, reducing photodamage to the sample. These capabilities are typically only afforded by lightsheet imaging modalities, but at the cost of greatly increased system complexity and non-conventional imaging geometries.

## Data Availability

Data underlying the results presented and the code used for image reconstruction and hardware control can be found at the GitHub repository [14].

## Supplemental document

See Supplement 1 for supporting content.
