# Supplementary material for: Fast, multicolour optical sectioning over extended fields of view with patterned illumination and machine learning
Source: Biomed Opt Express. 2024 Jan 25;15(2):1074–88. doi: 10.1364/BOE.510912 (PMC10890859; doi:10.1364/BOE.510912)
Supplement: Supplementary file 1 [file boe-15-2-1074-s001.pdf]

Supplemental Document

Biomedical Optics EXPRESS

Fast, multicolour optical sectioning over extended fields of view with patterned illumination and machine learning: supplement

EDWARD N. WARD,[1,†] REBECCA M. MCCLELLAND,[1,†] JACOB R. LAMB,[1] ROGER RUBIO-SÁNCHEZ,[1,2] CHARLES N. CHRISTENSEN,[1] BISMOY MAZUMDER,[1] SOFIA KAPSIANI,[1] LUCA MASCHERONI,[1] LORENZO DI MICHELE,[1,2] GABRIELE S. KAMINSKI SCHIERLE,[1] AND CLEMENS F. KAMINSKI[1,*]

[1]*Department of Chemical Engineering and Biotechnology, University of Cambridge, Cambridge, CB3 0AS, UK*
[2]*fabriCELL, Molecular Sciences Research Hub, Imperial College London, London, W12 0BZ, UK*
[†]*The authors contributed equally to this work*
[*]*cfk23@cam.ac.uk*

This supplement published with Optica Publishing Group on 25 January 2024 by The Authors under the terms of the Creative Commons Attribution 4.0 License in the format provided by the authors and unedited. Further distribution of this work must maintain attribution to the author(s) and the published article's title, journal citation, and DOI.

Supplement DOI: https://doi.org/10.6084/m9.figshare.25027889

Parent Article DOI: https://doi.org/10.1364/BOE.510912

Fast, multicolour optical sectioning over extended fields of view with patterned illumination and machine learning: supplemental document

1. COMPARISON OF PHOTOBLEACHING BEHAVIOUR BETWEEN POINT SCANNING CONFOCAL MICROSCOPY AND ML-OS-SIM

An advantage of machine learning optical sectioning structured illumination microscopy (ML-OS-SIM) over the widely used point scanning confocal technique is that imaging can be performed more quickly and at lower illumination intensities, resulting in less photodamage to the sample. To quantify this, two volumes of the same fixed Vero cell sample were imaged with similar imaging parameters and the reduction in the image brightness over time measured. Illumination intensity was chosen to achieve similar image quality, as judged visually. Other parameters were kept the same, as outlined in Table S1. Results are shown in Fig. S1.

Table S1. **Table of imaging parameters for comparing ML-OS-SIM and confocal microscopy**

Parameter	Value
Objective lens	UPLSAPO60XW, Olympus
Field of view	$44 \times 44\,\mu m^2$
Pixels	512×512
Volume (X × Y × Z)	$44 \times 44 \times 10\,\mu m^3$
Z sampling	$0.5\,\mu m$
Pixel size	86 nm

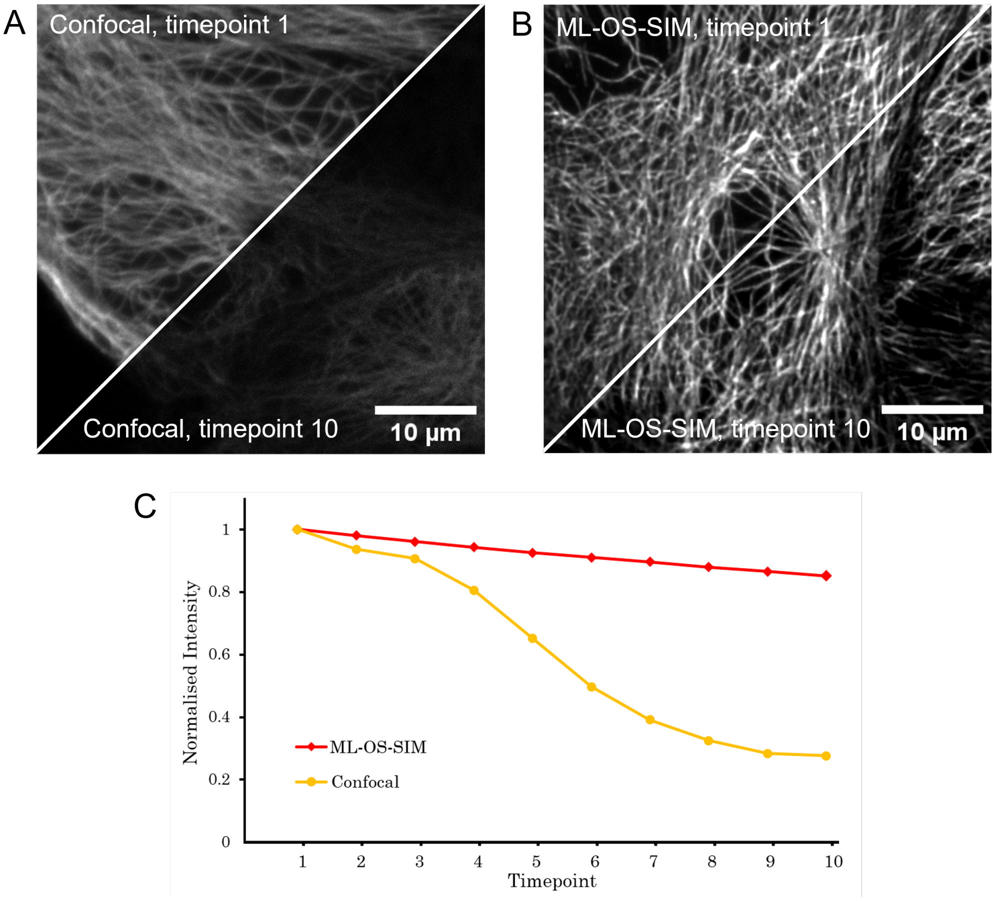

Fig. S1. Machine learning optical sectioning structured illumination microscopy (ML-OS-SIM) reduces photobleaching compared to confocal microscopy. A: 3D projections of the first and tenth timepoints when imaging a 44 $\mu m \times 44\,\mu m \times 10\,\mu m$ volume using confocal microscopy. Images show significant bleaching over time. B: Reconstructed 3D projections of the first and tenth timepoints when imaging the same size volume using ML-OS-SIM. The brightnesses of the images are proportional to the brightness of the raw data, and show minimal bleaching over time. C: The normalised brightness of the images as a function of the timepoint or number of volumes imaged. Confocal shows significantly faster bleaching owing to the higher laser power and longer imaging time required. A comparison of subfigures A and B shows that similar imaging performance was obtained using the imaging parameters set for the two methods. Images are of immunostained β-tubulin in fixed Vero cells, illuminated with $\lambda = 561$ nm light. Scale bars = 10 μm.

2. OPTICAL ALIGNMENT METHODOLOGY FOR ML-OS-SIM

Alignment can be optimised using a monolayer of sub-diffraction-sized fluorescent beads on a glass coverslip (Fig. S2). The two primary checks that need to be performed are the periodicity of the excitation fringes and the alignment of the beamlets at the back aperture of the objective lens.

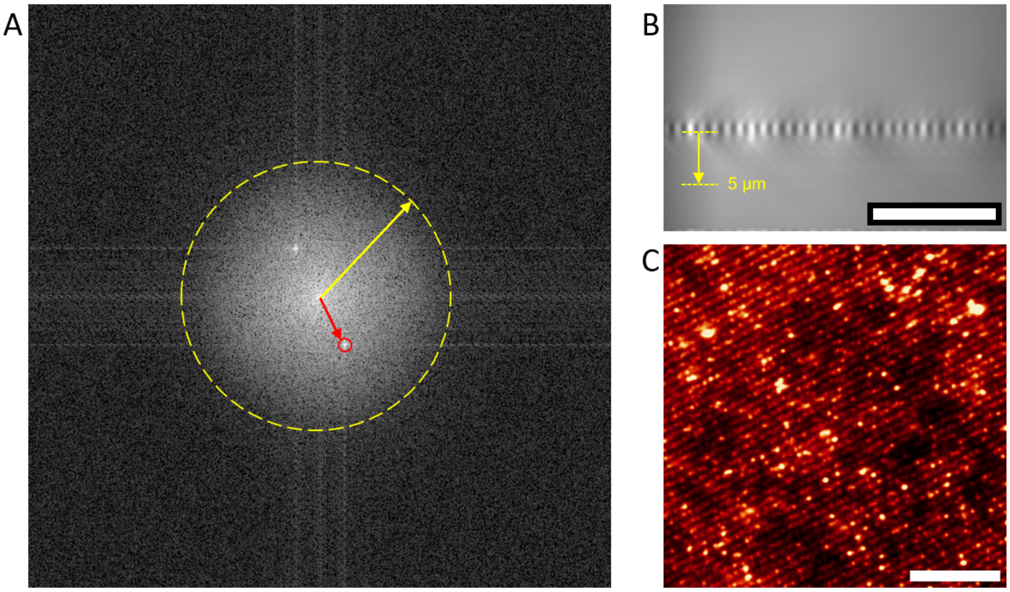

Fig. S2. System alignment checks. A: The periodicity of the stripes can be optimised by visualising the Fourier transform of a raw OS-SIM frame. For best performance, the spatial frequency of the pattern must be half of the cutoff. This can be confirmed in the Fourier transform by ensuring that the distance of the peaks from the centre (red arrow) is half the radius of the supported frequency cutoff (yellow arrow). B: x, z projection of a z-stack taken through a bead monolayer. Off-axis alignment of the excitation beams will result in change in pattern phase as the height of the sample is changed. In a misaligned system, this results in tilted fringes when the 3D volume is viewed in the x, z direction. Scale bar = 5 μm. Yellow dashed line indicates a 5 μm displacement from the focal plane. C: OS-SIM stripes visualised on a fluorescent bead monolayer. Both alignment checks can be carried out on the same sample if the bead density is optimised. Sub-diffraction beads must be sufficiently sparse to enable visualisation of the optical transfer function (A) while also being sufficiently dense to visualise the fringe pattern in the x, z projection. Scale bar = 5 μm.

3. GRAPHICAL USER INTERFACE

Image acquisition was controlled by software written in *Python*, which displayed a graphical user interface (GUI) for easy and intuitive use (Fig. S3). The software can control the camera exposure and readout, stage movement and scan mirror movement, and enables real-time viewing of reconstructed data via the GUI. Code is available in a GitHub repository [1].

The functionalities of the frames in the GUI shown in Fig. S3 are as follows. "Start/Stop Live Imaging": Simple start/stop buttons to begin and end the various processes of the system such as image acquisition, reconstruction and display. Relevant buttons in the "Saving" frame are enabled once live imaging has begun. "Saving": images can be saved in various ways using the buttons in this tab. "Start saving" will begin saving whichever image is currently being shown and continuously save images until it is pressed again. The button is relabelled "Stop saving" whilst saving is in process so this is clear to the user. In the case of 3D imaging, it will wait until the beginning of the volume to start saving, so that an integer number of stacks are recorded. "Snap shot" captures a single image and "Save stack" a single volume. In all cases, both the raw and the reconstructed images are saved in separate files, as well as a metadata file which contains relevant parameters such as which lasers are on, laser power, exposure time, time interval between captures, and, for 3D imaging, positions in z. The folder and filename to save images to can be selected by clicking the relevant buttons, which opens a dialog for straightforward selection of the directory. "Laser Control": In this frame, the lasers are turned on and off by clicking the On/Off buttons and the power can be selected using the drop-down menus. "ML Reconstruction": parameters relating to the live ML reconstruction can be entered here. These are preset at appropriate values such that clear reconstructed images should be displayed without requiring this input from the user. "Colour Channels and FOV": this frame controls which pixels in the FOV of the camera are selected as the region of interest (ROI) and subsequently processed, displayed and saved. "Imaging Parameters": values can be entered by the user to control the exposure time and, in the case of 3D imaging, the height of the volume to be imaged and the number of slices along the z direction to capture. Video parameters will control how many time points will be saved and the interval between them when saving using the "Save Video" button in the "Saving" frame. Lasers will be automatically switched off during the interval between image captures to minimise photobleaching. "Display Brightness" allows the user to increase the relative brightness of different colour channels in the display using sliders. Default setting is equal brightness. The "Imaging mode" drop-down menu can be used to select between four options: 1) display of a Fourier transform of the image, which can be used to calibrate the stripe pattern, 2) no reconstruction, which displays the raw frames captured by the camera with no processing, so that the stripe pattern illuminating the sample is visible, 3) single slice reconstruction, which displays a ML-reconstructed image of one plane in the sample and 4) volume reconstruction, which displays a maximum intensity projection of all the reconstructed slices in the range defined by Z Min to Z Max. The "Show live images" checkbox allows the user to stop the display of images in the GUI, which can improve the image acquisition speed and reduce computational load, the "Save reconstructions" checkbox allows the user to choose whether the reconstructed images should be saved in addition to the raw data, and "Fast mode" can be selected for faster acquisition speed without any live processing.

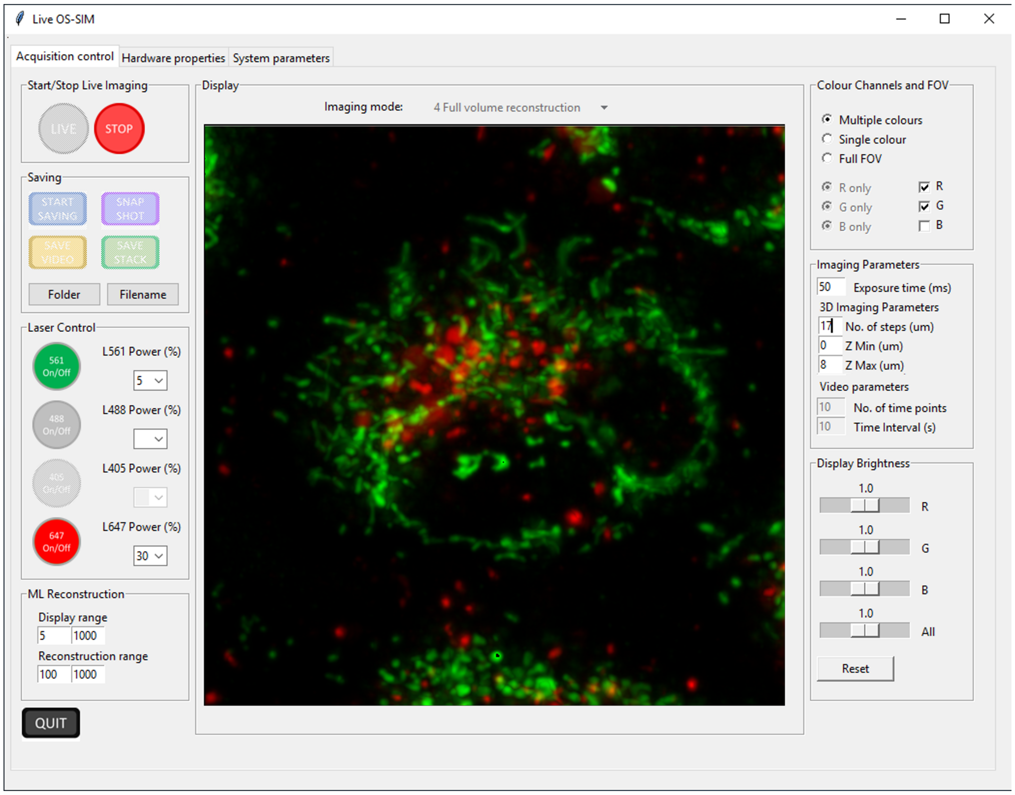

Fig. S3. Graphical user interface for acquisition, real-time processing and display of OS-SIM data.

4. IMAGE ACQUISITION AND PROCESSING SPEED

For high quality optical sectioning, the illumination pattern needs to have a high modulation depth and be static throughout each camera exposure. Once a single frame has been captured, the pattern phase is shifted with a small step of the scan mirror. Before the next frame can be acquired, the mirror must settle in the new position. Analogous to the switching time on spatial light modulator (SLM) systems, this step response time caps the maximum achievable frame rate with interferometric pattern generation. To assess this limit, we made use of the rolling shutter of the camera to measure the time taken for the mirror to complete a single pattern phase shift. By triggering the galvanometric mirror step change to coincide with the start of a frame acquisition, the movement of the mirror can be seen as warping of the striped pattern during the first lines of the frame readout (Fig. S4). In this way, the settle time, t_{settle}, can be calculated from the number of lines, N, before the pattern stabilises:

$$t_{settle} = N \times t_{line}, \tag{S1}$$

where t_{line} is the line time of the camera. Images were captured of the illumination pattern over a $11 \times 11\,\mu m^2/128 \times 128$ pixel FOV with 561 nm illumination and a 0.2 ms exposure time (Fig. S4B). Using the 11.4 μs line time of the camera, the settle time was calculated to be 207 μs, and was confirmed by introducing a delay between the galvanometric mirror step and the camera frame trigger (Fig. S4C). This leads to a cap in the maximum possible acquisition speed of 4.8 kHz, corresponding to an imaging speed of 1.6 kHz. We note that in the current optical setup, the clear aperture of the scan mirror used (Scanlabs, DynAXIS M) is considerably larger than required. The inertia associated with the large scanning element leads to the relatively high settle times seen here. By referring to the data available on alternative scan elements with a smaller clear aperture, we estimate that this settle time could be reduced to 75 μs offering a theoretical maximum acquisition speed of 13 kHz or an imaging speed of 4.4 kHz.

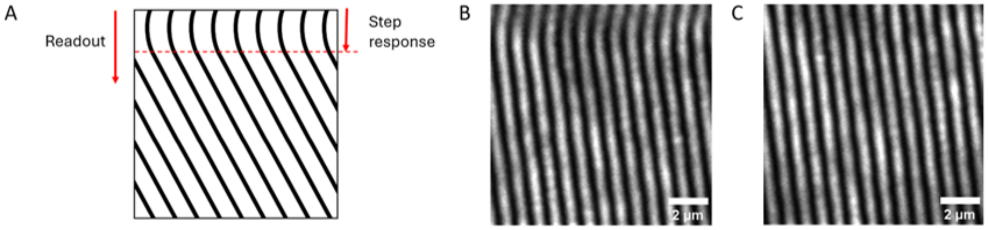

Fig. S4. Interferometric OS-SIM is limited by the settle time of the galvanometric mirror. A: Schematic of expected fringe distortion during galvanometric mirror movement. Bending can be seen at the top of the image as the phase of the pattern was changing whilst the first lines were read out from the camera. The settle time was calculated using Eq. S1. B: Representative illumination pattern measured with a 191 μs exposure and no delay between phase step and camera frame trigger. C: Representative illumination pattern measured with a 191 μs exposure and 207 μs delay. With this delay introduced, the galvanometric mirror is stable throughout frame exposure and no pattern warping is observed. Scale bars are 2 μm.

When the live reconstruction feature of the system is to be used, the maximum frame rate becomes limited by the rate at which images can be reconstructed and displayed live via the GUI. Images were captured over $44 \times 44\,\mu m^2$ with the minimum possible exposure time, 10 μs, to measure this. Reconstructed images could be displayed on the GUI in one colour and three colours at rates of 11 and 3 Hz, respectively, sufficient to enable live sample navigation and assessment of the image quality during imaging. Of the 94 ms processing time required per single-colour reconstructed image, 71 ms was required for acquiring and reading-in three raw images from the camera and 9 ms for the ML reconstruction itself.

5. SAMPLE PREPARATION

A. Bead monolayer

The monolayer was generated by air-drying 5 μL of 1:10,000 dilution of 20 nm Carboxylate-Modified Microspheres (ThermoFisher, F8786) onto a 175 μm coverglass at room temperature. Fluorescent beads were red-labelled with excitation/emission maxima 580/605 nm.

B. Live COS-7 cell culture

Cell culture COS-7 cells were obtained from ATCC (American Type Culture Collection, CRL-1651). These cells were grown in DMEM (Dulbecco's Modified Eagle's Medium, Sigma: D6546) supplemented with 10% heat inactivated FBS (Fetal Bovine Serum, Life Technology Invitrogen:10500064), 1% Antibiotic-Antimycotic mix (Invitrogen: 15240-062) and 2 nM Glutamax-1 (Life Technologies: 35050-038). The culture was maintained in T75 or T25 flasks at 37 °C temperature with 5% CO_2 atmosphere. Cells were maintained in the logarithmic phase of growth and passaged on reaching 65-75% confluence (twice weekly). For imaging, COS-7 cells were seeded into a Nunc Lab-Tek II chambered cover glass (Thermo Fisher Scientific, 12-565-335) and allowed to grow until 70% confluency.

Transfection & labelling COS-7 cells were transfected with 200 ng mCherry-PH plasmid (Addgene, 36075), using Lipofectamine 2000 transfection method as directed in the manufacturer's protocol. Cells were allowed to grow for a further 24 h before being subjected to imaging, to achieve optimum expression of PH domain of PLCdelta1. For labelling mitochondria, 100 nM of Mitotracker dye was added into the well from the stock of 100 μM solution and cells were incubated with the dye for 1 h before imaging. Imaging was performed with a stage-top incubator (OKOLab) at 37°C and 5% CO_2.

C. Fixed Vero cell culture

Vero cells (from monkey kidney tissue) were plated into 8-well plates (Ibidi), 20,000 cells per well, and cultured under standard conditions (37 °C, 5% CO_2) in minimum essential medium (Sigma Aldrich) supplemented with 10% foetal bovine serum (Gibco) and 2 mM L-lutamine (GlutaMAX, Gibco). After 24 h, cells were fixed by incubation with 4% methanol-free formaldehyde and 0.1% glutaraldehyde in cacodylate buffer (pH 7.4) for 15 min at room temperature, washed three times with PBS and then permeabilised by incubation with a 0.2% solution of saponin in PBS for 15 min. Unspecific binding was blocked by incubating with 10% goat serum and 100 mM glycine in PBS and 0.2% saponin for 30 min at room temperature. Without washing, the samples were incubated with the primary antibody (mouse anti-beta-tubulin: ab131205) diluted 1:200 in PBS containing 2% BSA (bovine serum albumin) and 0.2% saponin overnight at 4 °C. After three washes in PBS, the samples were incubated with the secondary antibody (goat anti-mouse conjugated to AlexaFluor568) diluted 1:400 in PBS containing 2% BSA and 0.2% saponin for 1 h at room temperature in the dark. Samples were then washed 3 times with PBS and imaged.

D. Preparation and re-organisation of DNA-functionalised Unilamellar Vesicles

D.1. Electroformation of de-mixed Giant Vesicles

Giant Unilamellar Vesicles were prepared with electroformation [2–4]. Briefly, indium tin oxide (ITO) slides underwent a cleaning routine of 15 min sonication cycles of Isopropanol followed by MilliQ water and subsequent drying under a gentle a nitrogen flow. Lipid films were generated on the conductive side of an ITO slide (heated to ~60 °C) by drop casting 45 μL of lipid mixture (DOPC/DPPC/Chol at a 2:2:1 molar ratio, 4 mg/mL) and gently spreading them with a glass coverslip. The slide was placed in a dry silica dessicator under vacuum for 1 h. Electroformation chambers were assembled using a ~ 1 mm thick polydimethylsiloxane (PDMS) spacer to couple two ITO slides enclosing approximately 400 μL of filtered sucrose buffer (300 mM). The chambers were connected to a frequency generator with clamps and subjected to a sinusoidal alternating current with voltage amplitude of 2 V with a frequency of 10 Hz for 2 h followed by 1 h at 2 Hz. Finally, vesicles were retrieved gently with a pipette and stored at room temperature in the dark to prevent photobleaching and photooxidation.

D.2. DNA nanostructures assembly

The responsive DNA nano-devices were adopted from previous work [5]. DNA oligonucleotides, purchased lyophilised (Integrated DNA Technologies [IDT], Eurogentec, and Biomers) and purified by the supplier with high-performance liquid chromatography (HPLC), were resuspended

in Tris-Ethylenediaminetetraacetic acid (EDTA) buffer (1× TE: 10 mM Tris + 1 mM EDTA, pH 8.0) to a final concentration of 100 μM. DNA nanostructures were subsequently self-assembled with a slow quenching temperature ramp (95 °C down to 20 °C at a rate of -0.5 °C·min^{-1}) on a thermal cycler (Alpha Cycler 2 PCRMax) in a buffer containing 1× TE +100 mM NaCl. Assembled nanostructures were stored at 4 °C prior to usage.

D.3. Vesicle functionalisation with DNA nanostructures

Membrane attachment of DNA nanostructures was done following established functionalisation schemes [5–7].

Briefly, 9.2 μL of vesicles were added to a mixture of DNA nanostructures (16.7 μL) and 57.4 μL of a correcting buffer, resulting in iso-osmolar conditions containing 1× TE + 100 μM NaCl + 87 mM Glucose. DNA-GUV mixtures were left overnight under rotation at room temperature and in the dark to avoid photobleaching. During imaging, Fuel/Antifuel strands were added sequentially in iso-osmolar buffers at 10× excess with respect to the anchor modules and the fluorescent cargo to trigger the re-organisation of the vesicles, as shown schematically in Fig. S5.

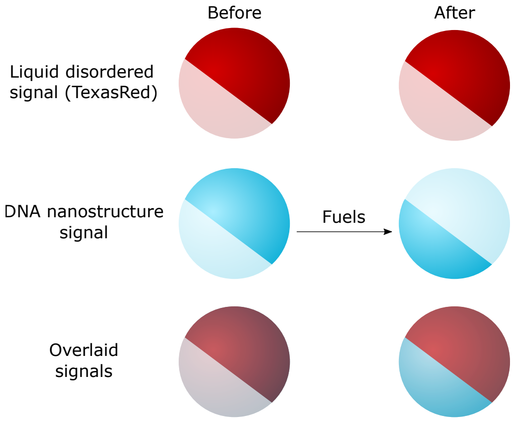

Fig. S5. Diagram to show the re-organisation of DNA-functionalised Giant Unilamellar Vesicles. The addition of chemical nucleic-acid signals (fuels) triggers the lateral partitioning of DNA nanostructures (blue) to the liquid-ordered phase. The liquid disordered phase (red) is stained with a fluorescent (TexasRed-DHPE) lipid marker. In the initial configuration, the overlapping DNA and lipid fluorescent signals showcase the preferential affinity of DNA nanostructures for liquid-disordered phases, while cargo transport relocates the fluorescent nanostructures to the liquid-ordered phase.

E. Sequence of DNA oligonucleotides used

The sequence of DNA oligonucleotides used is presented in Table S2.

Table S2. **Sequence of DNA oligonucleotides used**

Strand	Sequence 5′ 3′
Bbb,chol	/Cholesteryl-TEG/GTTGTGGTGGTGAGTGTG
Bb	CATCTCACTACTCAACACCACACTCACCACCACAAC
Memf	GTGTTGAGTAGTGAGATG/AlexaFluor488/
Bbb, toc	/Octyl-Tocopherol/GTTGTGGTGGTGAGTGTG
Bb, chol	CATCTCACTACTCAACACCACACTCACCACCACAAC/...
	Cholesterol-TEG/
Cb, chol	/Cholesteryl-TEG/CAATCACACCACAAACACCCAACACAACAAC...
	AAACC
Cbb, chol	GTGTTTGTGGTGTGATTG/Cholesterol-TEG/
Linf,*	GCTCTCTCATCACTAC/AlexaFluor488/
LinF12	GTGTTGAGTAGTGAGATGATTCGCGTAGTGATGAGAGAGCGTTGT...
	AGGTTTGTTGTTGTG
Fuel1	GTTGTTGTGTTGGCCTTACTTCACG
Antifuel2	GCGAATCATCTCACTACTCAACACTTCACCTCAAAC

REFERENCES

1. E. N. Ward, C. N. Christensen, and R. M. McClelland, "ML-OS-SIM," GitHub, https://github.com/edward-n-ward/ML-OS-SIM (2023).
2. M. I. Angelova, S. Soléau, P. Méléard, F. Faucon, and P. Bothorel, "Preparation of giant vesicles by external AC electric fields. Kinetics and applications," in *Trends in Colloid and Interface Science VI*, (Steinkopff, Darmstadt, 1992), pp. 127–131.
3. M. I. Angelova and D. S. Dimitrov, "Liposome electroformation," Faraday Discuss. Chem. Soc. **81**, 303 (1986).
4. S. L. Veatch and S. L. Keller, "Separation of Liquid Phases in Giant Vesicles of Ternary Mixtures of Phospholipids and Cholesterol," Biophys. J. **85**, 3074–3083 (2003).
5. R. Rubio-Sánchez, S. E. Barker, M. Walczak, P. Cicuta, and L. D. Michele, "A Modular, Dynamic, DNA-Based Platform for Regulating Cargo Distribution and Transport between Lipid Domains," Nano Lett. **21**, 2800–2808 (2021).
6. R. Rubio-Sánchez, B. M. Mognetti, P. Cicuta, and L. Di Michele, "DNA-Origami Line-Actants Control Domain Organization and Fission in Synthetic Membranes," J. Am. Chem. Soc. **145**, 11265–11275 (2023).
7. D. Morzy, R. Rubio-Sánchez, H. Joshi, A. Aksimentiev, L. Di Michele, and U. F. Keyser, "Cations Regulate Membrane Attachment and Functionality of DNA Nanostructures," J. Am. Chem. Soc. **143**, 7358–7367 (2021).
